# High-Resolution Micro-CT for Morphologic and Quantitative Assessment of the Sinusoid in Human Cavernous Hemangioma of the Liver

**DOI:** 10.1371/journal.pone.0053507

**Published:** 2013-01-07

**Authors:** Jinghao Duan, Chunhong Hu, Hua Chen

**Affiliations:** 1 College of Biomedical Engineering, Tianjin Medical University, Tianjin, China; 2 Department of Hepatobiliary Surgery, Tianjin Medical University Cancer Hospital, Tianjin, China; The Chinese University of Hong Kong, Hong Kong

## Abstract

Hepatic sinusoid plays a vital role in human cavernous hemangioma of the liver (CHL), and its morphologic investigation facilitates the understanding of microcirculation mechanism and pathological change of CHL. However, precise anatomical view of the hepatic sinusoid has been limited by the resolution and contrast available from existing imaging techniques. While liver biopsy has traditionally been the reliable method for the assessment of hepatic sinusoids, the invasiveness and sampling error are its inherent limitations. In this study, imaging of CHL samples was performed using in-line phase-contrast imaging (ILPCI) technique with synchrotron radiation. ILPCI allowed clear visualization of soft tissues and revealed structural details that were invisible to conventional radiography. Combining the computed tomography (CT) technique, ILPCI-CT was used to acquire the high-resolution micro-CT images of CHL, and three dimensional (3D) microstructures of hepatic sinusoids were provided for the morphologic depiction and quantitative assessment. Our study demonstrated that ILPCI-CT could substantially improve the radiographic contrast of CHL tissues in vitro with no contrast agent. ILPCI-CT yielded high-resolution micro-CT image of CHL sample at the micron scale, corresponding to information on actual structures revealed at histological section. The 3D visualization provided an excellent view of the hepatic sinusoid. The accurate view of individual hepatic sinusoid was achieved. The valuable morphological parameters of hepatic sinusoids, such as thrombi, diameters, surface areas and volumes, were measured. These parameters were of great importance in the evaluation of CHL, and they provided quantitative descriptors that characterized anatomical properties and pathological features of hepatic sinusoids. The results highlight the high degree of sensitivity of the ILPCI-CT technique and demonstrate the feasibility of accurate visualization of hepatic sinusoids. Moreover, there is a correlation between the CHL and the size or morphology of hepatic sinusoids, which offers a potential use in noninvasive study and analysis of CHL.

## Introduction

**Figure 1:**
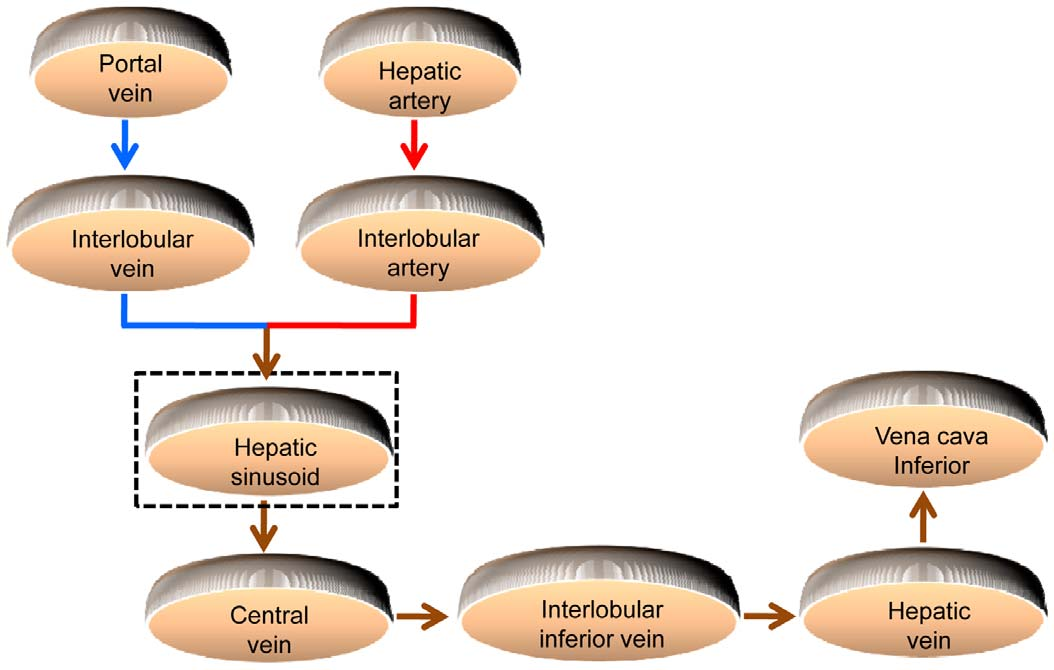
Illustrative presentation of the hepatic microcirculation system. The blue lines indicate the venous blood, the red lines indicate arterial blood, and the brown lines indicate the mixed blood. The blood is mixed in the hepatic sinusoids, where the metabolic activity and exchange of nutrients, oxygen etc. with the hepatocytes takes place.

Cavernous hemangioma of the liver (CHL) is the most common benign solid tumor of the liver [1]–[3]. Macroscopically, the tumor is distinguished by some cavernous and honeycomb textures. Microscopically, it is composed of some abnormal and dilated hepatic sinusoids with different sizes and various shapes [4]. The hepatic sinusoids are circuitous and show labyrinth-like structure. In the normal liver, the sinusoids are irregular, saccular or tubular, whose diameters range from 20 to 30 microns, and they are separated from hepatocytes by the perisinusoidal space. The hepatic sinusoids and hepatocytes are the primary structures of the hepatic lobule. As shown in Figure 1, portal venous blood rich in nutrients is delivered to the interlobular vein, while hepatic arterial blood, rich in oxygen, is delivered to the interlobular artery. Blood from these two afferent vessels combines and ows through the network of the sinusoids between the liver cell cords [5]. Subsequently, the mixed blood drains radially towards the central veins, and the central veins cluster towards the outflow interlobular inferior vein and hepatic veins [6]. Finally, the blood flows into the vena cava inferior. The slow blood flow in hepatic sinusoids creates possibilities for full materials exchange between the plasmas and the hepatocytes, which is helpful to the function of the hepatocyte in producing and detoxicating. Additionally, the hepatic sinusoids also contain phagocytic cells of the mononuclear phagocyte series known as Kupffer cells, which can immediately clear bacterias and virus coming from intestinal canal. Obviously, the hepatic sinusoids play an important part in the blood circulation and material exchange of the liver. In practice, CHL can alter the morphology of hepatic sinusoids, and the pathological features of CHL are always associated with the size or morphology of hepatic sinusoids. A study on morphology and structure of the hepatic sinusoid is beneficial to learn the blood circulation and material exchange in CHL, and it also conduces to characterize the formation, development and variation of CHL.

Conventionally, histological section has been used to provide the morphologic and quantitative assessment of the hepatic sinusoid. However, the invasive technique has some inherent limitations such as its destructiveness, complexity and sampling error. Accurate visualization of the hepatic sinusoids has been limited by the resolution and contrast available from existing imaging techniques such as conventional radiography, ultrasound, computed tomography (CT) and magnetic resonance imaging (MRI). Thus, there is a need for reliable, sensitive and noninvasive imaging techniques for assessing anatomical and pathological features of hepatic sinusoids.

X-ray phase-contrast imaging (PCI) is a new imaging technique that exploits a contrast mechanism based on differences in the x-ray refractive index distribution of a sample [7]–[9]. PCI can detect minute changes of density, and may provide much higher sensitivity than absorption for biological soft tissues with small absorption differences, revealing soft tissues discrimination at micrometer-scale resolution. Especially at the interface of tissues, where the refractive indices changes significantly, a distinct edge enhancement effect is shown in the PCI image, thus producing an improved image quality. In-line phase-contrast imaging (ILPCI) is a holography technique which generates intensity distribution including phase information [9]–[12]. In comparison with other PCI techniques, ILPCI has one unique advantage of a particularly simple experimental setup requiring no optical element. ILPCI computed tomography (ILPCI-CT) can provide substantially enhanced contrast in combination with the PCI advantage, promising to provide high-resolution micro-CT images, especially for soft tissues. Combining the three dimensional (3D) visualization technique, ILPCI-CT can acquire 3D morphology of inner microstructures in biomedical samples without contrast agents, which avoids unpleasant side effects of the contrast agent. Moreover, 3D visualization can overcome the limitations of the planar radiography mode that suffers from the structural overlapping of tissues. Precise 3D visualization provides an excellent means of analyzing and characterizing anatomical and pathological features of soft tissues in various medical applications and reveals structural details that are invisible to conventional radiography. In recent years, ILPCI-CT technique has attracted wide attention, and exhibits the excellent sensitivity over conventional absorption-based CT when imaging soft tissues, such as liver, brain, lung, kidney etc. [13]–[20]. Previous studies indicate that the high-resolution 3D visualization can improve detection of diseases, especially for early detection and diagnosis of various diseases [18]–[20]. As a nondestructive 3D technique with high-resolution, ILPCI-CT is suitable for 3D morphological research of complex microstructures inside the biomedical sample, and thus provides new possibilities for the morphologic depiction and quantitative evaluation of the hepatic sinusoid.

In this paper, ILPCI technique was used to image different degrees of human CHL samples without contrast agents, and 3D microstructures of CHL tissues were visualized using ILPCI-CT technique. The morphology and structure of hepatic sinusoids were investigated. Some valuable quantitative assessments characterizing anatomical properties and pathological features of hepatic sinusoid, such as thrombi, diameters, surface areas and volumes of hepatic sinusoids in CHL, were performed. The purposes of this study were to explore whether ILPCI-CT technique could render high-resolution 3D visualization of CHL tissues and could permit the accurate visualization for morphologic and quantitative assessment of the hepatic sinusoid.

## Materials and Methods

### Samples

This study was approved by the ethics committee of Tianjin Medical University, Tianjin, China, and written informed consent (as outlined in PLOS consent form) was obtained from all patients. Different degrees of human CHL samples were prepared in the Department of Hepatobiliary Surgery, Tianjin Medical University Cancer Hospital. The samples were cut into small pieces of 5 mm×3 mm with a height of approximately 8 mm and fixed in 10% buffered formalin prior to imaging. During CT imaging, the samples were sealed in cylindrical polyethylene containers with 8 mm in diameter. After imaging the samples were imbedded in paraffin, the HE staining histological sections (about 4 µm thick) were examined by light microscopy, and the images of the sections were digitized and stored. The histological section and its corresponding analysis were accomplished by an experienced pathologist. Histological findings served as the reference standard for interpretation of the CT images of the CHL samples.

### Principle of ILPCI

The behavior of x-ray while traversing a sample can be described by the complex refractive index
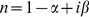
(1)where the real part 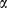 incorporates the refractive effects and results in a phase shift, while the imaginary part 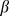 describes the absorption. In the x-ray energy range used in biomedical imaging, the phase-contrast term 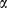
can be up to 1000 times greater than the absorption term 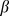. This implies that the PCI may provide much higher sensitivity than absorption-based imaging, especially for biological soft tissues, where absorption differences are very small.

ILPCI relies on Fresnel diffraction in free space of the wave field exiting the sample. It allows x-ray beams to propagate a sufficient distance away from the sample so that the phase shifts in the downstream beams are transformed into observable changes in the intensity distribution. The intensity variations are recorded by the image detector, forming a phase contrast image. Based on the Fresnel diffraction theory and the wave front function [12], the intensity in the Fourier space is given by
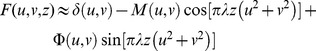
(2)where (*u, v*) is the coordinate in Fourier space, 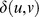
denotes the 2-D Dirac delta function, 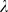 is the wavelength of the x-ray, 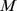
and 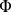 are the Fourier transform of 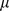 and 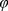, respectively, and *z* is the sample-to-detector distance (SDD). 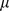 and 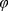 are the attenuation and phase shift induced by the object, and they are defined as:



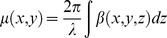
(3)

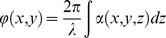
(4)where (*x, y*) is the spatial coordinates in the plane perpendicular to the propagation direction *z*. Obviously, Eq. (2) establishes a quantitative relationship between amplitude modulation and phase shift of the x-ray. There is a suitable SDD for given spatial frequency that corresponds to the detectable size, namely the resolution in the space domain. The optimal contrast is determined by the spatial frequency, wavelength, and SDD. For a weakly absorbing sample, the intensity distribution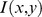 at 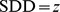 is approximated according to the transport of intensity equation (TIE):



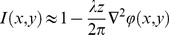
(5)Eq. (5) shows that the intensity in ILPCI is proportional to the Laplacian operator of the x-ray phase shift after passing through the sample. Variations in electron density of the sample lead to the changes of the phase, so tissue boundaries are enhanced by phase effects. Moreover, a second-order partial differential operator applied to the phase shift will greatly increase the image contrast, producing an enhanced edge contrast. The intrinsic edge enhancement allows the characteristic emphasis of boundaries, rendering tissue surfaces particularly well without the necessity for complex image processing.

### Image Acquisition

**Figure 2:**
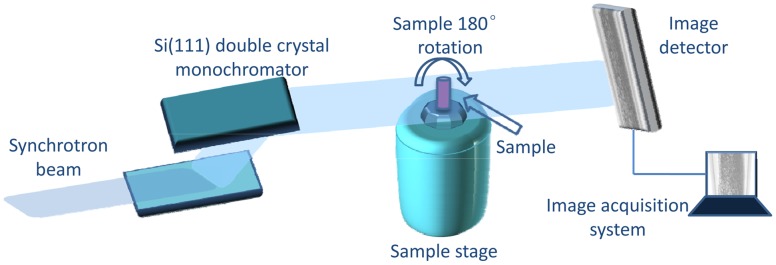
The schematic sketch of the experiment setup. Monochromatic synchrotron x-ray beam is projected on a sample mounted on a rotation sample stage, and the transmitted beam is recorded by an image detector. For tomographic scans, the samples can be rotated within 180° to record the projection images at different angles.

The experiments were performed at x-ray imaging and biomedical application beamline (BL13W1) of Shanghai Synchrotron Radiation Facility (SSRF) in China. The tunable energy range of the x-ray beam was 8–72.5keV, and the x-ray beam energy in the experiments was set at 16keV. The detector employed an x-ray CCD camera system with 3232×479 pixels, and 9×9 µm^2^ per pixel. The detector can be placed downstream of the objects at 0 cm to 8 m, and the distance between the samples and the CCD was 1 m in our experiment. The experiment setup mainly consisted of a Si(111) double-crystal monochromator, an automatic rotation sample stage and an x-ray sensitive CCD detector (Figure 2). In ILPCI imaging system, the incident white synchrotron x-ray beam emerging from the accelerator was monochromatized by a double-crystal monochromator in a symmetrical arrangement. Subsequently, the monochromatic beam passed through the object, and the transmitted beam was measured by a detector, producing an image. During the CT data acquisition, the samples were mounted on the rotation sample stage that was controlled by a precise step motor. A total of 1200 projection images of the sample were acquired when the samples was rotated within 180°. The exposure time per projection image was 75 ms. In addition, 20 flat field images (with no sample in the beam) were recorded to normalize the image intensity, and 10 dark field images, which were recorded when no photons hit the detector, were also collected to correct for the detector’s dark current offset [21]. Projection images were then reconstructed using the filtered back projection (FBP) algorithm.

### Image Reconstruction and 3D Visualization

**Figure 3:**
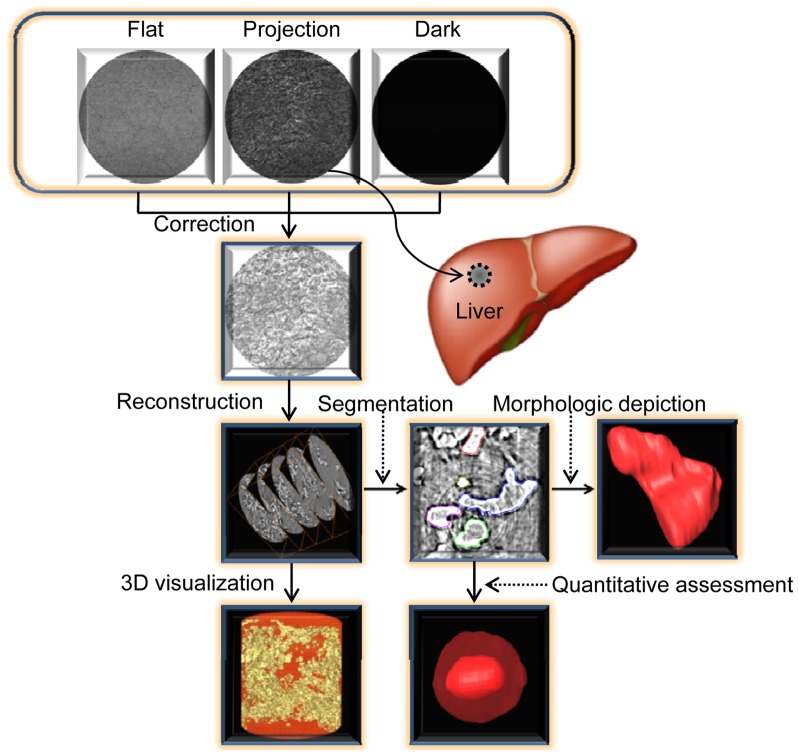
The schematic sketch of the image reconstruction and 3D visualization. The CT reconstruction was performed after the dark-field and flat-field corrections. Then, 3D visualization of CHL presented high-resolution morphology of the hepatic sinusoids, and the hepatic sinusoid was segmented to allow morphologic and quantitative assessment.

Figure 3 illustrated the schematic sketch of the image reconstruction and 3D visualization. In order to avoid massive data and save computation time, the regions of interest were firstly selected from the projection images, and they were stored as the images of 592×466 pixels. Then, the images were performed flat-field and dark-field correction, and the image smoothing method was utilized to suppress noise. Finally, the CT images were reconstructed using a standard FBP algorithm. Moreover, image enhancement was used to highlight the edge of the hepatic sinusoid after ring artifact correction. The 3D microstructures of CHL were visualized using the Amira 5.2 software, which allowed a clear visualization of the anatomical and pathological features of hepatic sinusoids. In addition, image segmentation was an essential step to achieve accurate 3D model. In our work, the segmentation method, which was based on human-computer interaction, was applied to separate the hepatic sinusoid from the background. The image segmentation method effectively guaranteed segmentation accuracy, and greatly improved the operation speed.

## Results

### Planar X-ray ILPCI Imaging

**Figure 4:**
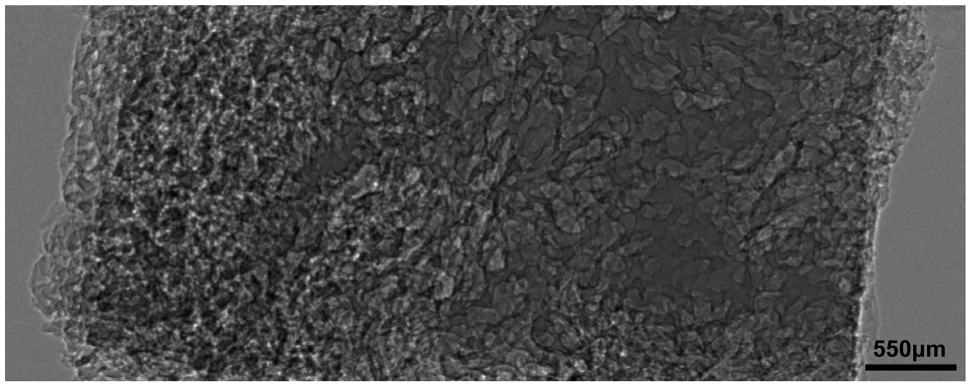
A planar x-ray image of the CHL sample. The hepatic sinusoids are clearly detected, but all of them are overlapped with each other.

Figure 4 is a planar image obtained from a CHL sample using x-ray ILPCI technique. The produced image contrast is related to changes in the x-ray refractive index of the tissues, and it results in extraordinary clarity compared with conventional x-ray images based on absorption effects. The image clearly displays the dilated hepatic sinusoids, as shown in Figure 4. Hepatic sinusoids with the diameter of less than 100 microns are detected. However, the quantitative measurements of hepatic sinusoids are hardly obtained due to overlapping of tissues.

### ILPCI-CT Imaging and Histopathologic Analysis

**Figure 5:**
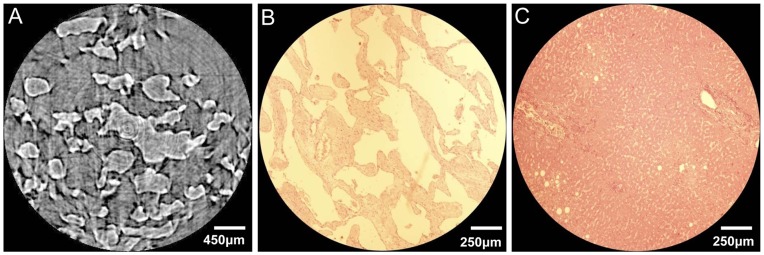
The CT image and histological section. (A) One slice of ILPCI-CT reconstruction images. (B) Histological section of the same CHL sample as in (A) (HE staining. Original magnification×40). (C) Histological section of a normal human liver (HE staining. Original magnification×40).

Planar imaging with ILPCI may not be suitable for hepatic sinusoids due to overlapping of structures. ILPCI-CT can be used to overcome this limitation, and it enables acquisition of an accurate 3D visualization of hepatic sinusoids inside the CHL sample. Fig. 5A is a CT image of the sample, and it depicts large and varied lacunas of hepatic sinusoids. Some hepatic sinusoids are nearly circular and isolated. Conversely, many hepatic sinusoids are irregular, characterizing by tortuous and anastomosing canaliform shapes. Figure 5B describes the histological section from the same sample, and it can serve as the references for interpretation of the CT reconstruction results. As shown in Figure 5A and Figure 5B, The ILPCI-CT image shows a resemblance to optical image of stained histological section, which confirms the correspondence of the CT finding with the morphology of the samples. Note that the unclear slicing angle and position of the histological section lead to mismatches between the Figure 5A and Figure 5B. As a comparison, the histological section from normal human liver is presented in Figure 5C. The normal hepatic sinusoids are radially arranged from the center of the central vein, and anastomose mutually to form a reticulation. Moreover, the diameters of normal hepatic sinusoids are much smaller compared with the hepatic sinusoids in CHL.

### 3D Microstructure Visualization of Different Stages of CHL Samples

**Figure 6:**
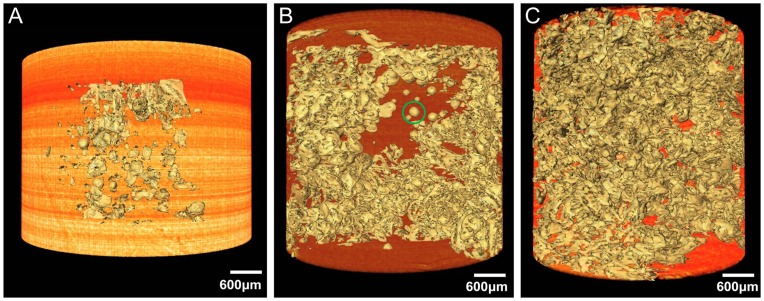
3D visualization of different stages of CHL samples. The 3D microstructure in mild, moderate and severe degrees CHL tissues are provided in (A), (B) and (C), respectively. The corresponding 3D model, shown in Video S1, S2 and S3 respectively, can be rotated in real time to observe the microstructures of hepatic sinusoids.

Combining surface rendering with volume rendering, 3D visualization of different stages of CHL samples are presented with vivid shapes and stereoscopic effects. The microstructures of hepatic sinusoids, which come from mild, moderate and severe degrees CHL tissues, are shown in Figure 6A, Figure 6B and Figure 6C respectively, and the associated animation are provided in supplementary material (See Video S1, S2 and S3 respectively). The 3D animation can be rotated within 360° to facilitate a clear 3D microstructures visualization of hepatic sinusoids. By comparison, it appears that the greater degree of lesion, the severer dilation of hepatic sinusoids. The dilated hepatic sinusoids in the severe CHL sample almost occupy the whole tumors.

**Figure 7:**
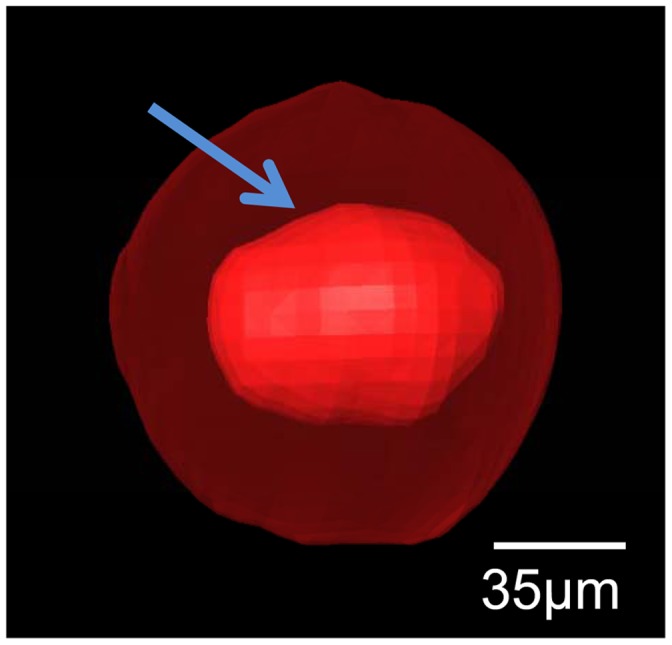
The thrombus in a hepatic sinusoid. The inner red part pointed by arrow is a thrombus. The outer transparent part is the wall of the hepatic sinusoid.

After 3D reconstruction, the inner microstructures of the hepatic sinusoids can be also acquired in computer by use of different view angles, which provide a good way to research the hepatic sinusoids of CHL samples. In our work, thrombi are observed in hepatic sinusoids. Figure 7 is the amplified image of the hepatic sinusoid indicated in the green circle region in Figure 6B, and it focuses on the display of the thrombus in a hepatic sinusoid. The value based on the ratio between the thrombus volume and the hepatic sinusoid volume is 16.86%. There are many influences predisposing to thrombus formation. Most of all, the collagen fiber of the hepatic sinusoid endothelium in CHL is heavily hyperplastic, which results in occlusion and fibrosis of the lacunas, and thrombus formation. The acute nature of some symptoms related to CHL may not be related to increase in size but rather to thrombosis and infarction of part of the tumor. The permeability of the hepatic sinusoid walls enhances as thrombus further develops. Moreover, the rise in the hydrostatic pressure may further give rise to hemorrhage for the hepatic sinusoid.

### Quantitative Assessment on the Morphology of Hepatic Sinusoids

**Figure 8:**
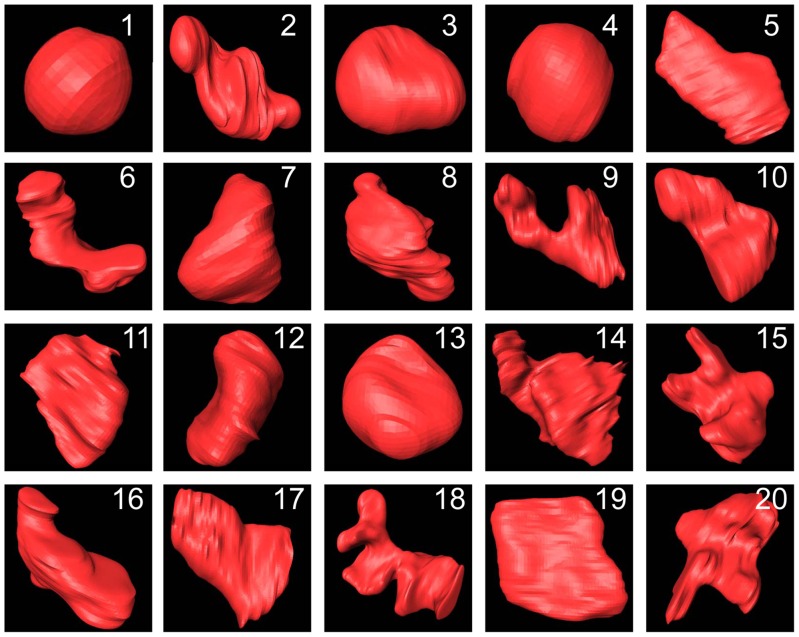
The 3D visualization of twenty single hepatic sinusoids. They were randomly chosen from different part of three CHL samples. The 3D visualization shows vivid shapes and stereoscopic effects. All the hepatic sinusoids are not of uniform size, and they are presented with various shapes.

**Figure 9:**
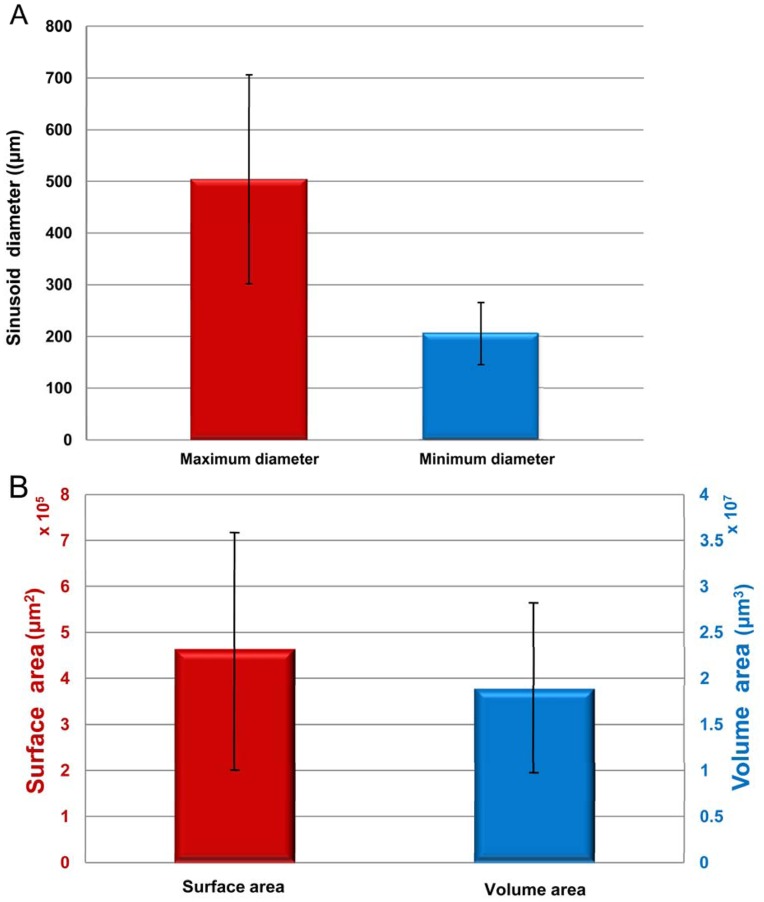
The statistics of the quantitative measurements shown in Figure S1. The mean values and standard deviations of the maximum and minimum diameters of the hepatic sinusoids are presented in (A), and the corresponding values of surface areas and the volumes are shown in (B).

The quantitative measurements of hepatic sinusoids are essential for us to understand the CHL. The diameters of the normal human hepatic sinusoids range from 20 to 30 µm. The normal human hepatic sinusoids have small diameters and large number, and the blood velocities in the hepatic sinusoids are very slow. These characteristics help to increase contact areas and prolong contract time between the hepatic sinusoids and the hepatocytes, and ensure that the hepatocytes can obtain sufficient nutriments with no metabolite accumulations. In this study, twenty single hepatic sinusoids are randomly chosen and visualized from 3D visualization of CHL (Figure 8). The minimum diameter, maximum diameter, surface area and volume of each hepatic sinusoid are measured, as summarized in supplementary material (See Figure S1). The results show that the diameters of the hepatic sinusoids in CHL range from 118.44 to 877.14 µm. Additionally, as shown in Figure 9A, the hepatic sinusoids average 205.66±60.10 µm and 504.08±202.13 µm in the minimum and maximum diameter, respectively. Compared with the normal hepatic sinusoids, the hepatic sinusoids in CHL are distinctly dilated with concave, convex or other irregular shapes. The dilated hepatic sinusoids compress seriously the hepatic cords. At the same time, the hepatic cords narrow and the hepatocytes are contractible, which give rise to scarce oxygen and nutriment supply to the hepatocytes and accumulational metabolites. Finally, the dilated hepatic sinusoids may cause atrophy, necrosis and fibrous tissue hyperplasia of the hepatocytes. In practice, the changes of surface areas and volumes in the hepatic sinusoids are closely related to the development of CHL. The surface areas of the normal human hepatic sinusoids range from 5×10^3^ to 1.5×10^4^ µm^2^, and the corresponding volumes range from 3×10^4^ to 1.5×10^5^ µm^3^. The surface areas of the hepatic sinusoids in CHL are 4.60×10^5^±2.60×10^5^ µm^2^ and range from 4.60×10^4^ to 9.45×10^5^ µm^2^, and the corresponding volumes are 1.88×10^7^±9.30×10^6^ µm^3^ in the range from 9.13×10^5^ to 4.16×10^7^ µm^3^, as provided in Figure 9B and Figure S1. As such, the surface areas and volumes of hepatic sinusoids in CHL increase significantly in comparison with the normal human hepatic sinusoids. With the increase of the surface area and volume, it is advantageous for the blood to enter into the lumen of the hepatic sinusoid, but this also speeds up the blood flow to some extent. The result is adverse to the absorption of nutriment for the hepatocyte, and may give rise to degeneration, atrophy and necrosis of the hepatocyte.

## Discussion

CHL represents the most common benign liver neoplasm, often being incidentally detected with asymptomatic [22]. However, numbers of giant CHL may be symptomatic by abdominal distension and discomfort. A part of CHL may be symptomatic by thrombocytopenia or hypofibrinogenemia. Some CHL, associating with spontaneous rupture and bleeding [23] or acute inflammatory process [24], have been reported. The hepatic sinusoids, as important structures of liver, play an important role in CHL. Generally speaking, the formation, development and variation of diseases always correlate with the changes of the structure and morphology. In particular, the clear visualization of tissue microstructures may improve early detection and accurate diagnosis of the diseases. Currently, the microstructure research of the hepatic sinusoid mainly relies on histological section, which is observed under the optical or electronic microscope. Nevertheless, histological section is an invasive method, and various limitations are evident such as unrepeatable use and intra-observer variation in histological interpretation. In contrast, noninvasive imaging techniques have inherent advantages in CHL studies. Available imaging techniques have improved significantly over the past decade. However, these techniques are not sensitive enough to detect the microstructures of hepatic sinusoids due to the restrictions of their spatial resolution and contrast. Thus, there is a need for new, sensitive and noninvasive diagnostic technique, which can reveal more detailed microstructures about hepatic sinusoids or even replace histological section.

PCI, as a novel imaging technique, has opened new avenues for medical diagnostics owing to its unique spatial and contrast resolution in the past few years, and it permits clear visualization of soft tissues with the weak absorption characteristics. As the simplest PCI technique, ILPCI has been proven to allow clear structures of biological samples. Combining the CT technique, ILPCI-CT can acquire the high-resolution micro-CT images. Our present study demonstrates that ILPCI-CT can substantially improve the radiographic contrast of CHL tissue in vitro without any contrast agent, and provide a valuable tool in the 3D microstructure visualization of hepatic sinusoids. The technique to obtain an accurate 3D morphology of CHL tissues is especially crucial to investigate anatomical and pathological features of hepatic sinusoids, which may facilitate the study of microcirculation mechanism and pathological changes of CHL. In this study, high-resolution micro-CT images of CHL samples are obtained, and histological sections confirms the diagnostic relevance and value of ILPCI-CT technique. Also, qualitative description and quantitative evaluation of hepatic sinusoids, such as thrombi, diameters, surface areas and volumes, provide objective and quantitative indexes for the morphology of human CHL, which may be beneficial for clinician to make an objective decision on the CHL disease. In addition, this investigation is convenient for us to understand the microcirculation of CHL and the hemodynamic changes in the hepatic sinusoid. Moreover, the ILPCI-CT technique may have a potential use in the study of CHL complications, such as thrombocytopenia, hypofibrinogenemia and acute inflammatory, which are associated with the morphology and thrombus formation of hepatic sinusoids.

ILPCI technique shows great promise for biomedical imaging because of improved contrast. Quantitative phase information in ILPCI holds great potential in improving delineation of soft tissue structures. In practice, phase retrieval methods may be employed to extract quantitative phase information from the raw ILPCI images [21], [25]. Moreover, the phase retrieval tomography is particularly suited for the 3D visualization of biological soft tissues [17], [26], and it may significantly improve visualization of anatomical and pathological features in the CHL sample. Additionally, the minimization of radiation exposure has been a hot issue for CT imaging study in biomedical samples, and it may become a key requirement in many imaging applications. While ILPCI-CT significantly ameliorates soft tissues contrast, radiation dose continues to be an issue in biomedical applications. In recent years, high-quality images can be reconstructed from far less data or measurements based on the compressive sampling (CS) theory. The CS theory opens the possibility to reconstruct image from drastically fewer projections than the Nyquist-theorem demands [27]. Combining CS theory with ILPCI-CT technique, high-resolution micro-CT image of soft tissues can be presented using substantially reduced projection data compared with the traditional FBP algorithm, which significantly reduce possible radiation doses while maintaining the advantages of ILPCI-CT [28].

There are some limitations while the preliminary results of this study are encouraging. First, the study lacks a certain degree of statistical significance due to small number of samples, and more different degrees of human CHL samples need be further investigated in order to achieve a statistically relevant difference in ILPCI-CT images. Now, the corresponding work is in progress. Second, as discussed above, the normal human hepatic sinusoids, whose diameters range from 20 to 30 microns, play a crucial role in blood circulation and materials exchange in liver. However, the spatial resolution of our study is not adequate for microstructures observation of hepatic sinusoids. The theoretical spatial resolution of PCI can be on the order of microns or even sub-microns, and thus further increase of spatial resolution can solve this issue. Third, to better highlight the high degree of sensitivity of ILPCI-CT technique, histological sections should be obtained at the same position at which the CT images have been acquired before. Finally, it would be very important to determine if this technique successfully enables in vivo observation in order to evaluate the ILPCI-CT value in clinical application of CHL. In addition, ILPCI technique requires a highly coherent and bright x-ray source, and the corresponding images are mostly acquired by using synchrotron radiation. Recently, conventional laboratory x-ray source is under development, and several novel techniques have been put forward [10], [29]. These researches demonstrate the potential of ILPCI in clinical environment, which may provide a sensitive noninvasive imaging technique for auxiliary diagnosis and analysis of CHL.

## Supporting Information

Figure S1 **The quantitative measurement of each hepatic sinusoid.** (A) The maximum diameter and the minimum diameter of each hepatic sinusoid. (B) The surface area and the volume of each hepatic sinusoid.
(TIF)

Video S1 **Animated view of the mild CHL sample.** This is the same 3D model shown in Figure 6A. The rotation of the model permits the viewers to better observe the mild CHL tissue from different angles, and facilitates an exact assessment of the hepatic sinusoid. In the mild CHL sample, the hepatic sinusoids are almost isolated.
(AVI)

Video S2 **Animated view of the moderate CHL sample.** This is the same 3D model shown in Figure 6B. By comparing with video S1, the hepatic sinusoids in video S2 are much more dilated and conglutinated with each other. There are some isolated hepatic sinusoids in the moderate CHL tissue.
(AVI)

Video S3 **Animated view of the severe CHL sample.** This is the same 3D model shown in Figure 6C. Comparing with the video S1 and video S2, the hepatic sinusoids in video S3 are much dilated. The dilated hepatic sinusoids almost occupy the whole tumor. There are few isolated hepatic sinusoids in the severe CHL tissue.
(AVI)
