# Supplementary figures and images for: High-Resolution Micro-CT for Morphologic and Quantitative Assessment of the Sinusoid in Human Cavernous Hemangioma of the Liver
Source: PLoS One. 2013 Jan 7;8(1):e53507. doi: 10.1371/journal.pone.0053507 (PMC3538536; doi:10.1371/journal.pone.0053507)

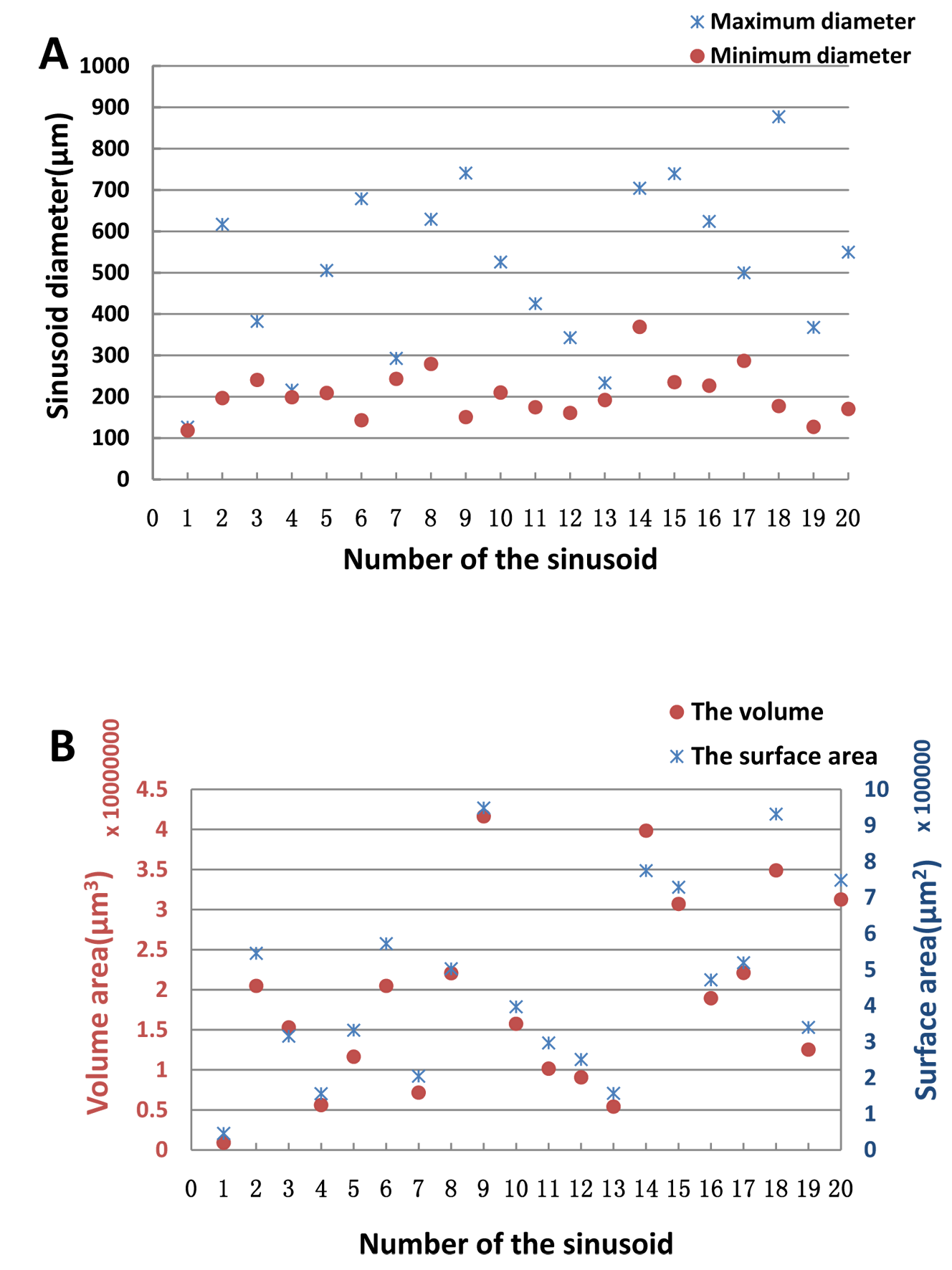

Supplement: Figure S1 — The quantitative measurement of each hepatic sinusoid. (A) The maximum diameter and the minimum diameter of each hepatic sinusoid. (B) The surface area and the volume of each hepatic sinusoid. (TIF) [file pone.0053507.s001.tif]
